# Supplementary material for: The causal role of circulating inflammatory markers in osteoporosis: a bidirectional Mendelian randomized study
Source: Front Immunol. 2024 Jul 18;15:1412298. doi: 10.3389/fimmu.2024.1412298 (PMC11291241; doi:10.3389/fimmu.2024.1412298)
Supplement: Supplementary file 1 [file DataSheet_1.zip › supplementary material/OSTEOPOROSIS-CIM/GCST90274847.tsv.gz_leave_one_out.pdf]

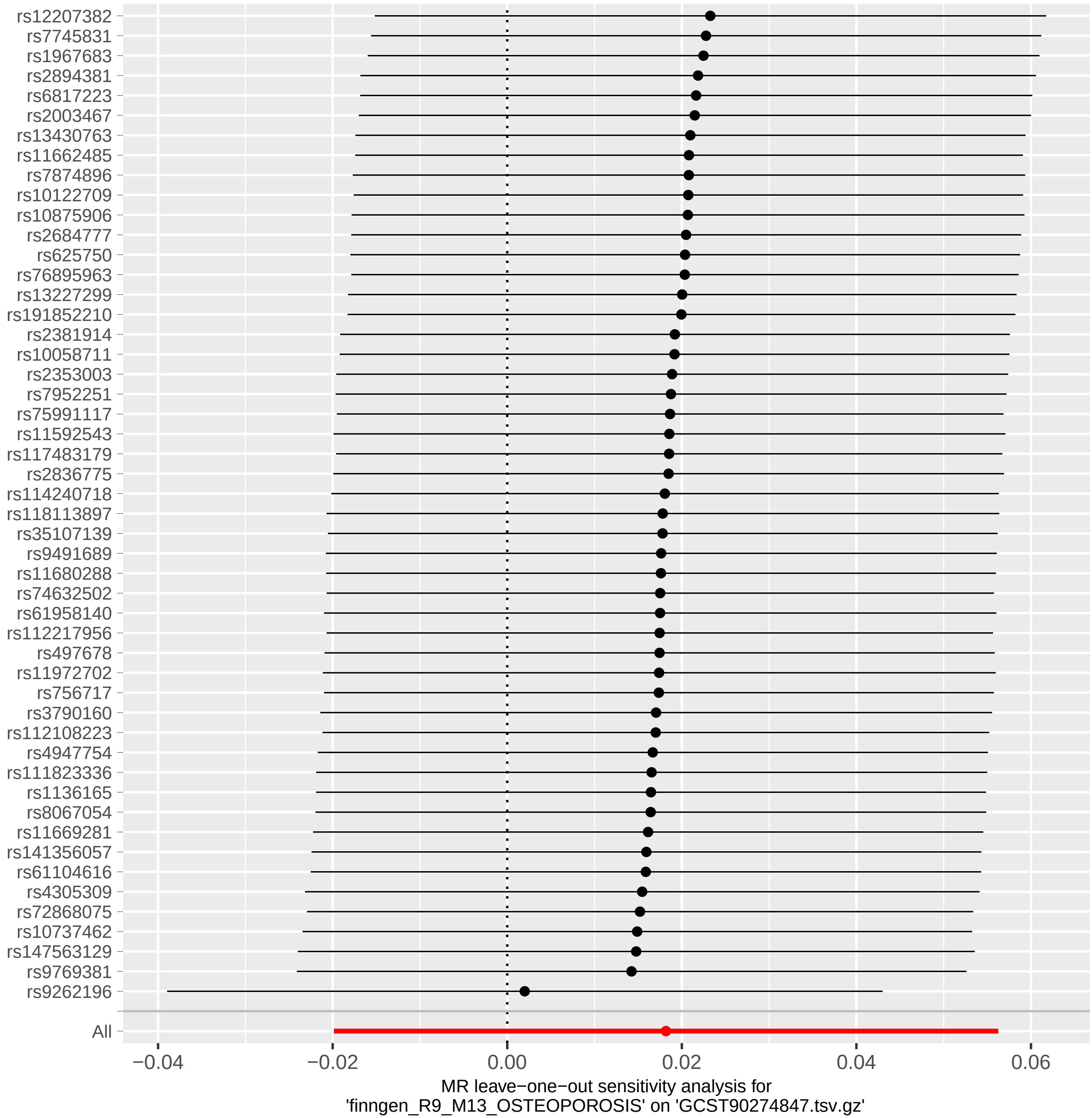
rs12207382
rs7745831
rs1967683
rs2894381
rs6817223
rs2003467
rs13430763
rs11662485
rs7874896
rs10122709
rs10875906
rs2684777
rs625750
rs76895963
rs13227299
rs191852210
rs2381914
rs10058711
rs2353003
rs7952251
rs75991117
rs11592543
rs117483179
rs2836775
rs114240718
rs118113897
rs35107139
rs9491689
rs11680288
rs74632502
rs61958140
rs112217956
rs497678
rs11972702
rs756717
rs3790160
rs112108223
rs4947754
rs111823336
rs1136165
rs8067054
rs11669281
rs141356057
rs61104616
rs4305309
rs72868075
rs10737462
rs147563129
rs9769381
rs9262196
All
−0.04
−0.02
0.00
0.02
0.04
0.06
MR leave−one−out sensitivity analysis for
'finngen_R9_M13_OSTEOPOROSIS' on 'GCST90274847.tsv.gz'
